# Parvovirus B19-Associated Hematological Complications: Two Case Scenarios of Pure Red Cell Aplasia and Hemophagocytic Lymphohistiocytosis

**DOI:** 10.7759/cureus.111355

**Published:** 2026-06-23

**Authors:** Nitesh Yadav, Jayashree D Kulkarni, Gayathri J, Anusha R

**Affiliations:** 1 Hemato-Oncopathology, Sri Shankara Cancer Hospital and Research Center, Bengaluru, IND; 2 Clinical Pathology, Birat Medical College Teaching Hospital, Budhiganga, NPL; 3 Pathology, Sri Shankara Cancer Hospital and Research Center, Bengaluru, IND

**Keywords:** bone marrow, giant pronormoblast, hemophagocytic lymphohistiocytosis, parvovirus b19, pediatric hematology, pure red cell aplasia

## Abstract

Parvovirus B19 (PVB19) has tropism for erythroid precursors and may cause clinically important hematological syndromes, including pure red cell aplasia (PRCA). Less commonly, it may be associated with secondary hemophagocytic lymphohistiocytosis (HLH). We report two PVB19-associated hematological presentations identified through bone marrow morphology. Case 1 was a 60-year-old woman with severe anemia, reticulocytopenia, and bone marrow erythroid hypoplasia. Giant pronormoblasts prompted PVB19 testing, and anti-PVB19 IgM was positive, consistent with PVB19-associated PRCA. Case 2 was a two-year-old girl with prolonged fever, hepatosplenomegaly, edema, ascites, and pancytopenia. Bone marrow examination showed hemophagocytosis and erythroid precursors with cytoplasmic blebs/pseudopodia. Hyperferritinemia and hypofibrinogenemia supported an HLH phenotype, and anti-PVB19 IgM was positive. Both patients received supportive care with corticosteroid-based immunomodulation; intravenous immunoglobulin was used as part of management. Hematological recovery was documented during follow-up. PVB19 should be considered in unexplained cytopenias when marrow morphology shows giant pronormoblasts, erythroid hypoplasia, or suggestive erythroid viral cytopathic changes. Early recognition may direct timely etiologic confirmation and individualized supportive or immunomodulatory treatment.

## Introduction

Parvovirus B19 (PVB19) is a small, non-enveloped, single-stranded DNA virus with a predilection for erythroid progenitor cells. Viral entry is mediated principally through the erythrocyte P antigen and related co-receptors, explaining its dominant effect on erythropoiesis [1,2]. In immunocompetent hosts, infection is often self-limited; however, in patients with reduced hematologic reserve, hemolytic disorders, or impaired immune clearance, PVB19 may produce severe anemia, transient aplastic crisis, or persistent pure red cell aplasia (PRCA) [1-3].

In immunocompromised individuals, including recipients of solid organ or hematopoietic stem cell transplantation, patients receiving chemotherapy, and those with underlying hematological disorders, impaired viral clearance may lead to persistent parvovirus B19 infection. In such settings, the virus may cause chronic anemia, PRCA, prolonged cytopenias, and occasionally severe systemic complications. Recognition of PVB19 infection in these vulnerable populations is important because early diagnosis and appropriate supportive or immunomodulatory therapy can result in favorable clinical outcomes.

PVB19-associated PRCA is characterized by severe anemia, reticulocytopenia, and marked reduction or absence of erythroid precursors in the marrow. Giant pronormoblasts with viral cytopathic changes are a classic clue and should trigger confirmatory serology or polymerase chain reaction testing when available [4,5].

PVB19 has also been reported as an infectious trigger for secondary hemophagocytic lymphohistiocytosis (HLH), a hyperinflammatory syndrome characterized by fever, organomegaly, cytopenias, and laboratory evidence of immune activation [6,7]. The present report highlights two contrasting PVB19-associated hematological presentations and emphasizes the diagnostic value of bone marrow morphology, which provided the initial clue to diagnosis in both cases before serological confirmation. Although parvovirus B19-associated pure red cell aplasia and HLH have been individually described in the literature, the simultaneous illustration of these contrasting hematological manifestations in a single report is uncommon.

## Case presentation

Case 1: PVB19-associated pure red cell aplasia

A 60-year-old woman presented with fatigue, exertional dyspnea, poor appetite, and approximately 8-kg weight loss over one year. Symptoms were insidious in onset and gradually progressive over one year. Fatigue and exertional breathlessness worsened over the preceding months, prompting hematological evaluation. Hemogram showed severe anemia, with hemoglobin of 4.0 g/dL, white blood cell count of 6,290/µL, and platelet count of 260 x 10³/µL. The peripheral smear showed dimorphic anemia, and the reticulocyte count was 0.8%. Iron studies were not suggestive of iron deficiency. Serum lactate dehydrogenase (LDH) was 249 U/L, and direct bilirubin was 0.11 mg/dL, findings that were not suggestive of ongoing hemolysis. The markedly reduced reticulocyte count (0.8%) favored impaired erythropoiesis rather than peripheral red cell destruction.

**Figure 1:**
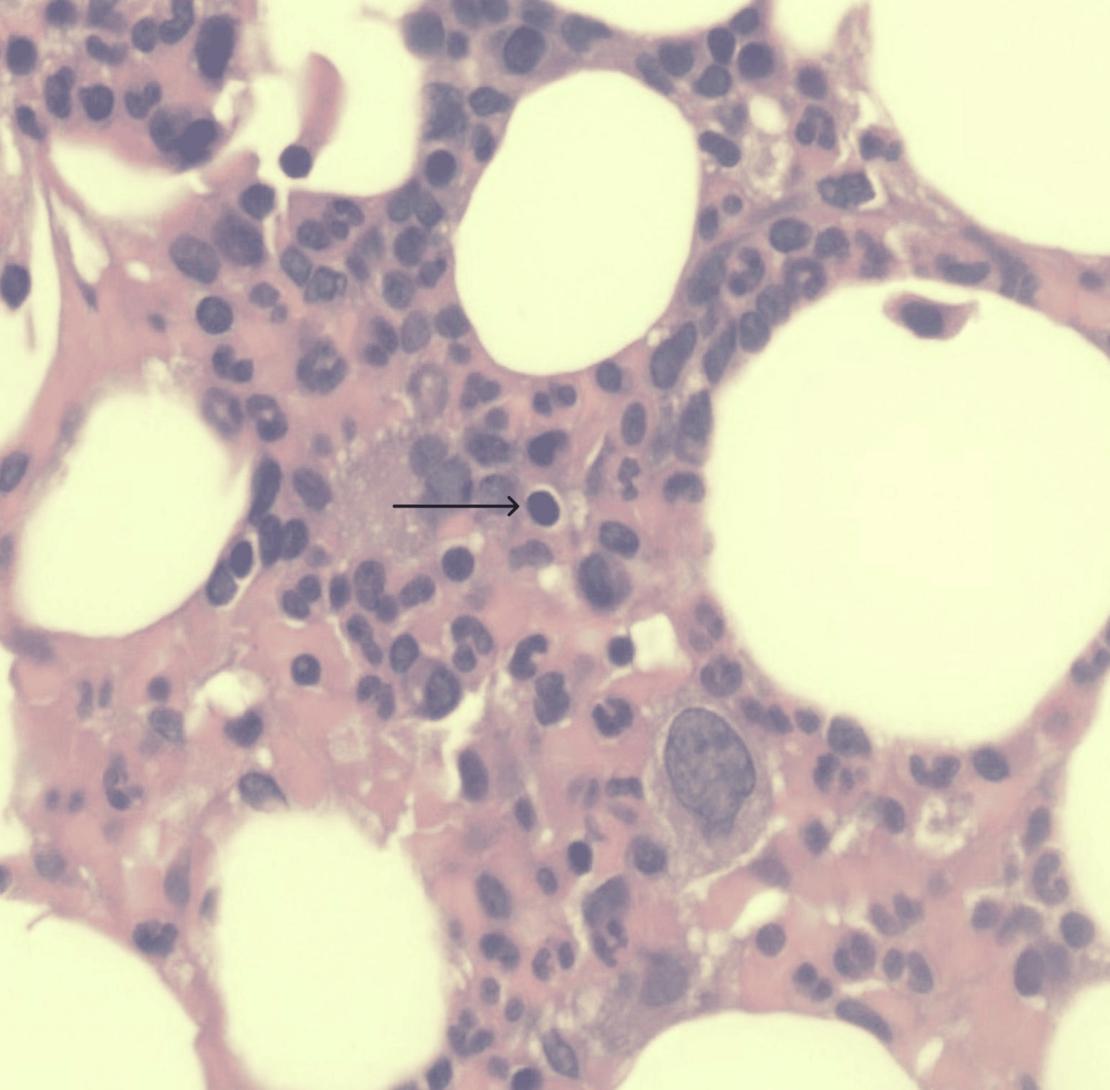
High-power view of the bone marrow biopsy from Case 1 showing marked erythroid hypoplasia. Hematoxylin and eosin stain, original magnification x100.

**Figure 2:**
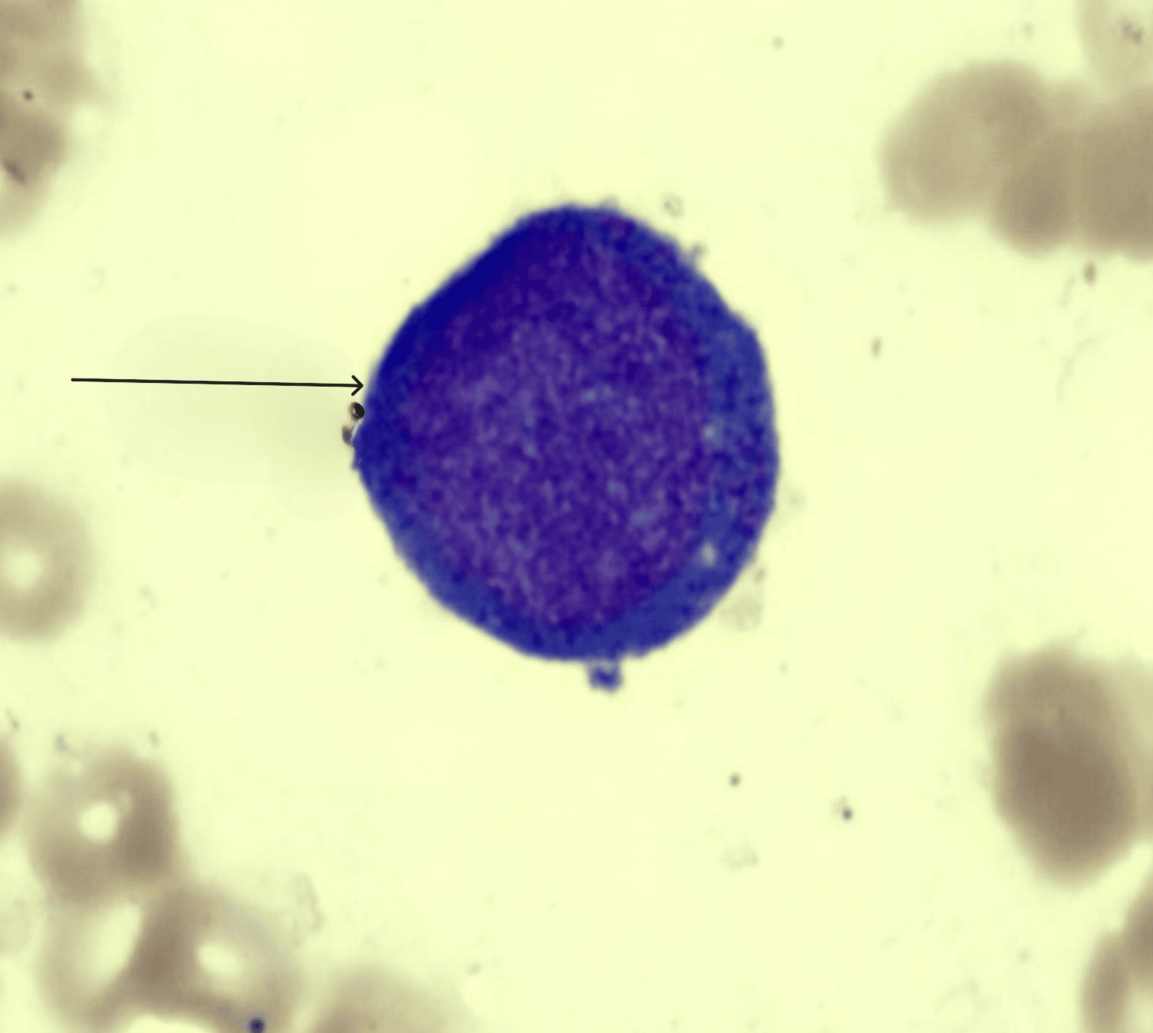
Bone marrow aspirate showing a giant pronormoblast in Case 1, supporting parvovirus B19-associated pure red cell aplasia. Wright-Giemsa stain, original magnification x100.

Bone marrow examination showed marked erythroid hypoplasia. Giant pronormoblasts were identified on the aspirate, raising suspicion for PVB19 infection (Figures 1-2). Anti-PVB19 IgM was positive, supporting recent PVB19 infection in the setting of PRCA.

The differential diagnosis included nutritional anemia, autoimmune PRCA, drug-related erythroid suppression, myelodysplasia, marrow infiltration, and viral-associated PRCA. The combination of severe reticulocytopenic anemia, erythroid hypoplasia, and PVB19-associated cytopathic morphology favored PVB19-associated PRCA.

The patient received red blood cell transfusion support and corticosteroid-based therapy; intravenous immunoglobulin (IVIg) was also used as part of management. Hemoglobin improved to 12.4 g/dL at six months.

Case 2: PVB19-associated HLH phenotype

A two-year-old girl presented with fever and progressive abdominal distension for two months. Fever was persistent throughout the illness and was followed by progressively increasing abdominal distension. During the weeks preceding admission, the child developed generalized edema and progressive enlargement of the liver and spleen. Clinical examination revealed hepatosplenomegaly and cannula-site ecchymosis. Hemogram showed pancytopenia, with hemoglobin of 4.3 g/dL, white blood cell count of 3,250/µL, and platelet count of 11,000/µL.

**Figure 3:**
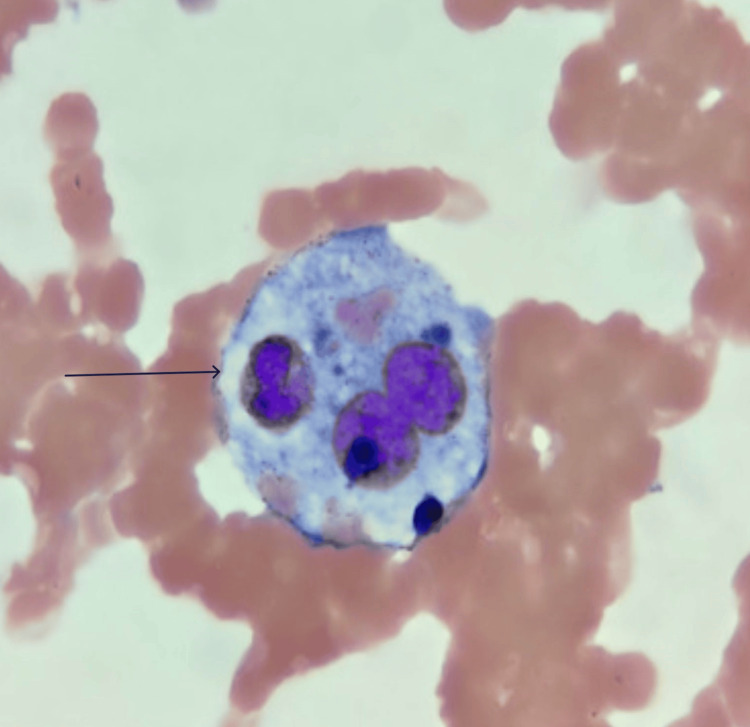
Bone marrow aspirate from Case 2, showing hemophagocytosis, with a histiocyte engulfing red blood cells. Wright-Giemsa stain, original magnification x100.

**Figure 4:**
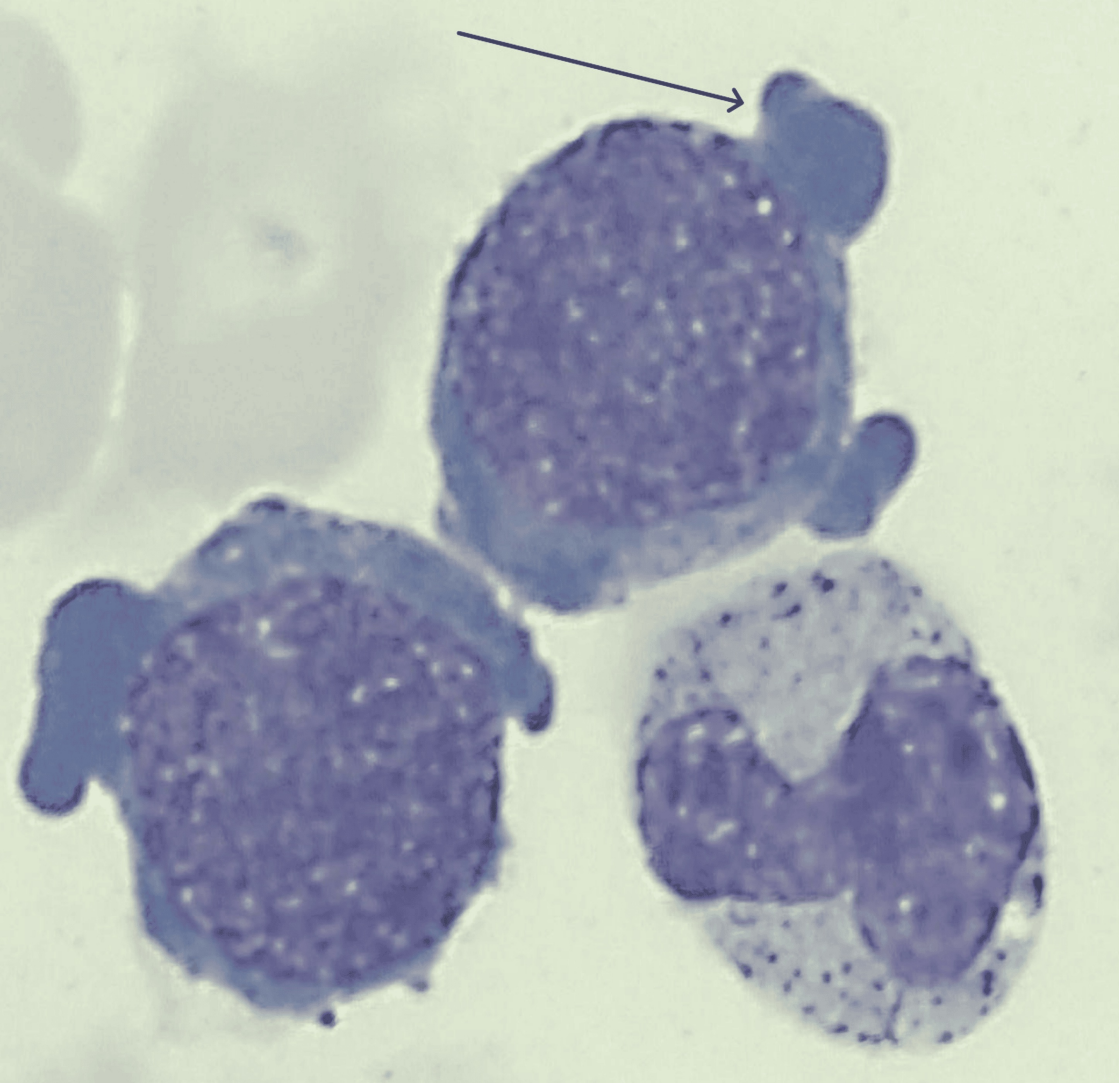
Erythroid precursors from Case 2, showing characteristic cytoplasmic blebs/pseudopodia described as "dog-ear" morphology, raising suspicion for parvovirus B19 infection. Wright-Giemsa stain, original magnification x100.

The bone marrow was normocellular, with erythroid hyperplasia, increased histiocytes, and hemophagocytosis (Figure 3). Erythroid precursors showed cytoplasmic blebs/pseudopodia described as "dog-ear" morphology (Figure 4), suggesting PVB19-related erythroid cytopathic change. Biochemical markers showed serum ferritin of 572 ng/mL, fibrinogen of 108.54 mg/dL, LDH of 624 U/L, and direct bilirubin of 7.42 mg/dL. Anti-PVB19 IgM was positive.

The differential diagnosis included severe infection or sepsis, malignancy-associated HLH, rheumatologic/macrophage activation syndrome, and primary HLH. The presence of fever, hepatosplenomegaly, cytopenias, hemophagocytosis, hyperferritinemia, and hypofibrinogenemia supported HLH, with PVB19 considered the likely associated trigger.

The child received supportive therapy and corticosteroid-based immunomodulation; IVIg-based therapy was also administered. Clinical and hematological improvement occurred during follow-up, with hemoglobin reported as 12.7 g/dL at the last follow-up. Table 1 presents the comparative clinical and laboratory findings in the two patients.

**Table 1:** Comparative clinical and laboratory findings in the two patients. HLH: hemophagocytic lymphohistiocytosis; LDH: lactate dehydrogenase; PVB19: parvovirus B19; PRCA: pure red cell aplasia; TLC: total leukocyte count

Parameters	Case 1 (PRCA)	Case 2 (HLH)
Age/sex	60 years/F	2 years/F
Duration of illness	1 year	2 months
Hemoglobin (g/dL)	4.0	4.3
TLC (/µL)	6,290	3,250
Platelets (/µL)	260x10³	11x10³
Reticulocyte count	0.8	NA
Peripheral smear	Dimorphic anemia	Pancytopenia with dimorphic anemia
Iron studies	Not suggestive of iron deficiency anemia	Not suggestive of iron deficiency anemia
Ferritin (ng/mL)	1531	572
Fibrinogen (mg/dL)	NA	108.54
LDH (U/L)	249	624
Bilirubin direct (mg/dL)	0.11	7.42
Bone marrow	Erythroid hypoplasia, giant pronormoblasts	Hemophagocytosis, dog-ear erythroblasts
PVB19 IgM	Positive	Positive
Final diagnosis	PVB19-associated PRCA	PVB19-associated HLH

## Discussion

HLH is a severe hyperinflammatory syndrome characterized by uncontrolled immune activation and cytokine dysregulation. In adults, early recognition of HLH and identification of the underlying trigger are essential for timely management and improved outcomes [8]. Consensus-based recommendations emphasize supportive care, corticosteroids, immunomodulatory therapy, and treatment of the precipitating cause in severe disease [9]. The HLH-2004 guidelines continue to provide the standard diagnostic framework using clinical, laboratory, and pathological criteria [10].

PVB19 infection is an uncommon, but important infectious trigger for secondary HLH and may also produce severe erythroid suppression with PRCA. In the present report, one patient demonstrated classical marrow findings of PRCA with giant pronormoblasts, while the second patient showed hemophagocytosis and characteristic erythroid cytopathic changes associated with an HLH phenotype.

Bone marrow examination played a critical role in both cases by identifying erythroid hypoplasia, viral cytopathic morphology, and hemophagocytosis, thereby facilitating early clinicopathological correlation. Serological confirmation, together with marrow morphology, supported the diagnosis of PVB19-associated hematological disease.

Management depends on disease severity and immune status. Supportive transfusions, corticosteroids, and IVIg therapy may result in favorable hematological recovery, particularly when diagnosis and treatment are initiated early. Both patients in the present report showed significant clinical and hematological improvement during follow-up.

Previous reports have described PVB19-associated PRCA and, less commonly, secondary HLH. Consistent with published literature, both of our patients responded favorably to supportive therapy and intravenous immunoglobulin. The present report highlights the diagnostic utility of characteristic bone marrow morphology in identifying these distinct manifestations of PVB19 infection.

## Conclusions

PVB19 infection may present with diverse hematological manifestations ranging from PRCA to secondary HLH. Careful bone marrow evaluation, recognition of characteristic erythroid cytopathic changes (including giant pronormoblasts, erythroid hypoplasia, and dog-ear erythroblasts), and appropriate serological testing are essential for diagnosis. In the present report, the patient with PRCA showed favorable hematological recovery following packed red cell transfusions and supportive management, while the patient with HLH demonstrated significant clinical improvement after prompt immunomodulatory therapy and corticosteroids. Both patients responded favorably to supportive therapy and IVIg. These cases emphasize that considering PVB19 infection in patients with unexplained cytopenias, PRCA, or HLH-like manifestations may facilitate timely diagnosis and appropriate management.
